# Mechanism of delayed seed germination caused by high temperature during grain filling in rice (*Oryza sativa* L.)

**DOI:** 10.1038/s41598-020-74281-9

**Published:** 2020-10-15

**Authors:** Chetphilin Suriyasak, Yui Oyama, Toshiaki Ishida, Kiyoshi Mashiguchi, Shinjiro Yamaguchi, Norimitsu Hamaoka, Mari Iwaya-Inoue, Yushi Ishibashi

**Affiliations:** 1 Faculty of Agriculture, Kyushu University, Fukuoka, 819-0395 Japan; 2 Institute for Chemical Research, Kyoto University, Uji, Kyoto 611-0011 Japan

**Keywords:** Plant physiology, Heat

## Abstract

High temperature during grain filling considerably reduces yield and quality in rice (*Oryza sativa* L.); however, how high temperature affects seed germination of the next generation is not yet well understood. Here, we report that seeds from plants exposed to high temperature during the grain filling stage germinated significantly later than seeds from unstressed plants. This delay remained even after dormancy release treatments, suggesting that it was not due to primary seed dormancy determined during grain filling. In imbibed embryos of heat-stressed seeds, expression of abscisic acid (ABA) biosynthesis genes (*OsNCEDs*) was higher than in those of control seeds, whereas that of ABA catabolism genes (*OsABA8′OHs*) was lower. In the aleurone layer, despite no change in GA signaling as evidenced by no effect of heat stress on *OsGAMYB* gene expression, the transcripts of α-amylase genes *OsAmy1C*, *OsAmy3B*, and *OsAmy3E* were significantly down-regulated in heat-stressed seeds in comparison with controls. Changes in promoter methylation levels were consistent with transcriptional changes of ABA catabolism-related and α-amylase genes. These data suggest that high temperature during grain filling results in DNA methylation of ABA catabolism-related and α-amylase gene promoters, delaying germination of heat-stressed seeds.

## Introduction

Environmental stresses, such as temperature, drought, salinity, and other abiotic stresses, strongly affect plant growth and development^1^. High temperature is one of the main environmental factors that cannot be avoided and cause losses in agricultural production worldwide. Ambient temperature strongly influences plant growth and development during both vegetative and reproductive stages^2^. Heat stress can shorten the period of grain development, resulting in insufficient grain filling and yield reduction in cereals^3,4^. Rice (*Oryza sativa* L.) is one of the most important crops for global food consumption, especially in Asia. It has been predicted that every 1 °C increase in average temperature would lead to about 10% reduction in rice yield^5^. Rice exposed to high temperature during grain filling develops chalky appearance, which reduces grain quality^6–9^. However, the effect of heat stress during grain filling on offspring growth and development remains to be elucidated.

Seed germination is the beginning of the plant’s life cycle. Seed dormancy and germination are strongly related to each other and are regulated by phytohormones, especially gibberellic acid (GA) and abscisic acid (ABA)^10^. After imbibition, GA biosynthesis and ABA catabolism are up-regulated to promote seed germination^11,12^. The transcription factor *GAMYB* induces α-amylase gene expression in a GA-dependent manner in the aleurone layers of barley, wheat, rice, and other cereals^13,14^. In response to GA from the embryo, *GAMYB* expression is induced and the encoded protein binds to the GARE boxes in the α-amylase gene promoters to induce α-amylase expression for starch degradation to fuel seed germination^14,15^. Environmental factors during seed development, especially temperature, strongly influence the level of primary dormancy^16^. A failure of a viable seed to germinate under favorable conditions is known as seed dormancy, which is controlled by several environmental factors such as light, temperature, and duration of seed storage^17,18^. In some barley (*Hordeum vulgare* L.) cultivars, moisture and low temperature during seed maturation are related to a lack of seed dormancy, resulting in preharvest sprouting^19^. In oilseed rape (*Brassica napus* L.), heat stress during seed filling decreases seed dormancy^20^. During seed development and maturation of *Arabidopsis* and wheat, temperature variations profoundly affect seed performance and dormancy^16,21^. In rice, high temperature during early endosperm development primes seed germination and seedling growth^22^.

The appropriate level of seed dormancy is a desirable trait for production of important cereal crops; understanding of how temperature during seed maturation affects seed dormancy and germination is necessary for crop production. Many studies have proposed the impacts of temperature during imbibition on seed germination^23–25^, but the effects of temperature during seed maturation have not been studied. In this study, we subjected rice plants to moderate heat stress from post-anthesis to harvest and show that high temperature during grain filling significantly delayed seed germination. We investigated the hormonal and epigenetic regulation of this phenomenon.

## Results

### Heat stress during grain filling significantly delays seed germination regardless of primary dormancy release

**Figure 1:**
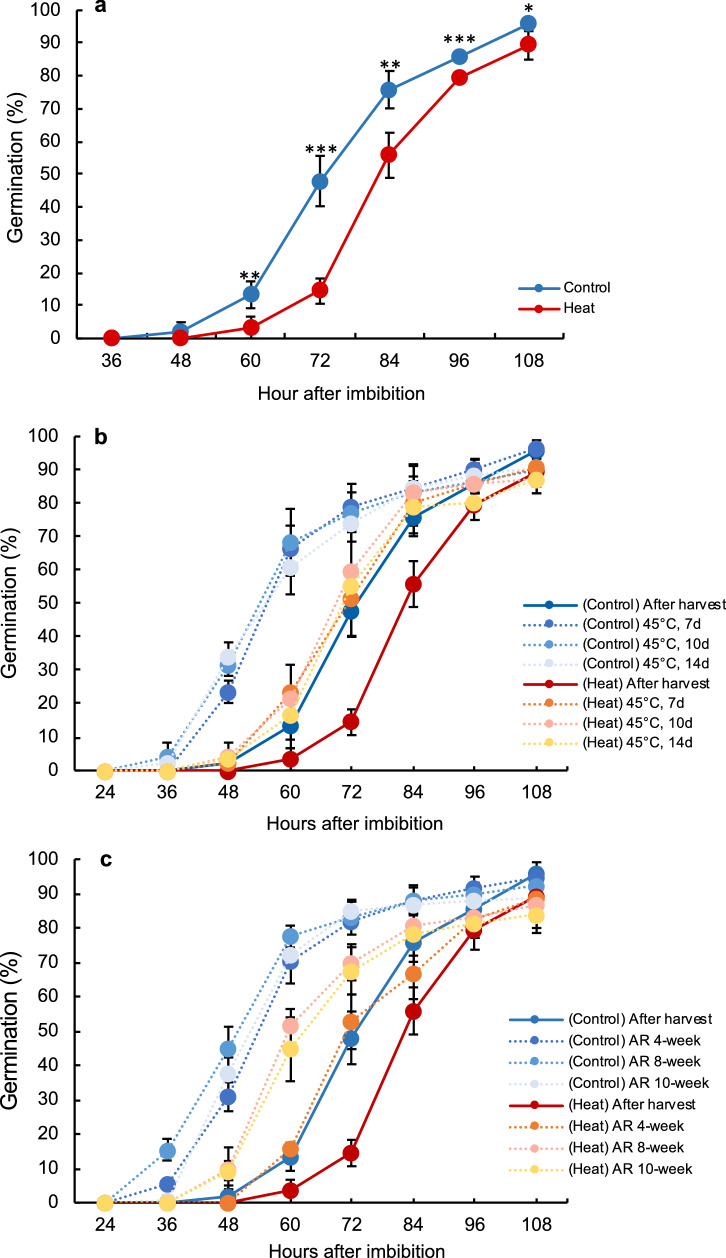
Delay in germination of seeds subjected to heat stress during grain filling stage. Germination rates of (**a**) freshly harvested seeds; (**b**) seeds subjected to 45 °C dormancy break treatments for 7, 10, or 14 days; and (**c**) seeds subjected to 26 °C after-ripening (AR) treatments for 4, 8, or 10 weeks. Significant differences are shown as *P* < 0.05*, *P* < 0.01**, and *P* < 0.001***, according to Student’s *t*-test (*n* = 3). Error bars, SD.

Seeds developed under heat stress germinated significantly later than control unstressed seeds (Fig. 1a). At 72 HAI (hours after imbibition), 48% of control seeds but only 14% of heat-stressed seeds germinated. Heat stress during grain filling did not affect seed viability, because germination rates of control and heat-stressed seeds at 108 HAI were similar. Similar delay was also observed in the other two cultivation years (Supplemental Fig. 1) and in other rice cultivars (Supplemental Fig. 2). To clarify whether this phenomenon was due to primary seed dormancy induced during grain development under stress, we subjected control and heat-stressed seeds to dormancy break treatment before germination test at 27 °C. Dormancy break treatment accelerated germination of both control and heat-stressed seeds (Fig. 1b). Although seed dormancy was released, control seeds still germinated faster than heat-stressed seeds. After-ripening treatment (26 °C) for 4, 8, or 10 weeks showed similar results (Fig. 1c). We calculated the average time required to reach 50% germination (*T*_50_) of seeds subjected or not to dormancy break or after-ripening treatment (Table 1). In both treatments, increasing treatment duration reduced *T*_50_ values, but *T*_50_ reached a plateau at 5 days (dormancy break) or 8 weeks (after-ripening treatment). Compared with after-harvest seeds, those subjected to dormancy-break or after-ripening treatment had lower *T*_50_ values regardless of heat stress; however, heat-stressed seeds still germinated significantly later in both treatments, as evidenced by significantly higher *T*_50_ values of heat-stressed seeds (Table 1). These results suggest that the delay in germination of heat-stressed seeds was not caused by primary dormancy.

**Table 1:** *T*_50_ values of after-harvest seeds and seeds subjected to dormancy-break or after-ripening treatment.

	Control	Heat	Student’s t-test
**Dormancy break**			
After-harvest	71.28 ± 3.34 a	79.72 ± 2.51 a	**
45 °C, 3 days	61.17 ± 2.58 b	73.70 ± 3.33 ab	***
45 °C, 5 days	56.70 ± 6.03 bc	69.67 ± 3.80 bc	**
45 °C, 7 days	57.86 ± 3.87 bc	67.42 ± 4.43 bc	**
45 °C, 10 days	51.22 ± 5.29 c	64.27 ± 8.25 c	*
45 °C, 14 days	55.74 ± 3.92 bc	69.20 ± 3.28 bc	***
**After-ripening**			
After-harvest	71.28 ± 3.34 a	79.72 ± 2.51 a	**
26 °C, 2-week	54.70 ± 2.96 b	68.27 ± 8.53 b	*
26 °C, 4-week	52.02 ± 2.52 b	69.04 ± 1.59 b	***
26 °C, 6-week	52.35 ± 4.85 b	60.95 ± 2.97 c	*
26 °C, 8-week	48.79 ± 2.19 b	57.83 ± 1.27 c	***
26 °C, 10-week	50.10 ± 3.92 b	58.57 ± 3.58 c	**

All seeds were germinated at 27 °C (*n* = 5 per condition).Within each column, values followed by different letters are significantly different (*P* < 0.05). Within each row, the significance of differences between control and heat-stressed seeds is shown according to Student’s *t*-test (*P* < 0.05*, *P* < 0.01**, and *P* < 0.001***). SD values are shown after *T*_50_ values.

### Changes in embryo ABA-metabolic gene expression and endogenous ABA content during seed germination

**Figure 2:**
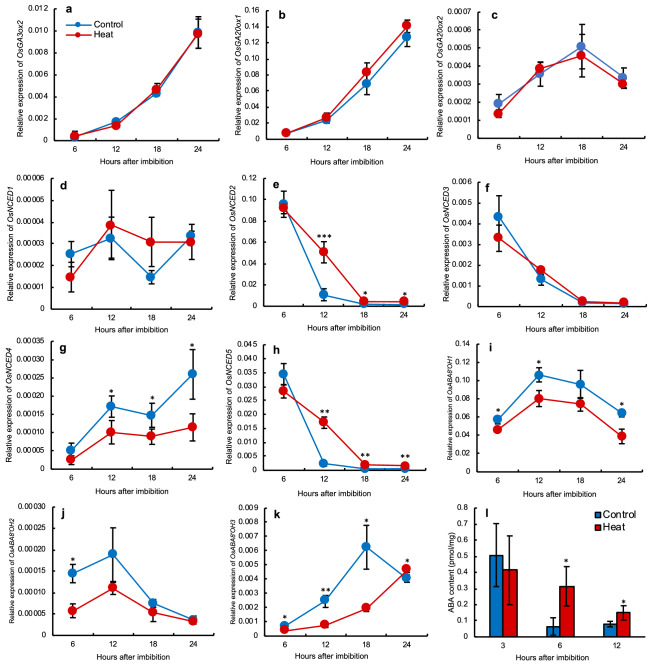
Relative expression of GA and ABA metabolism-related genes in imbibed embryos and endogenous ABA content. (**a**) *OsGA3ox2*; (**b**) *OsGA20ox1*; (**c**) *OsGA20ox2* (**d**) *OsNCED1*; (**e**) *OsNCED2*; (**f**) *OsNCED3*; (**g**) *OsNCED4*; (**h**) *OsNCED5*; (**i**) *OsABA8′OH1*; (**j**) *OsABA8′OH2*; (**k**) *OsABA8′OH3*; (**l**) endogenous ABA contents in embryos during imbibition. Significant differences are shown as *P* < 0.05*, *P* < 0.01**, and *P* < 0.001***, according to Student’s *t*-test (*n* = 3). Error bars, SD.

We examined the transcript levels of genes involved in the metabolism of GA and ABA, two major hormones regulating rice seed germination. In embryos, the expression of three GA biosynthesis genes, *OsGA3ox2*, *OsGA20ox1*, and *OsGA20ox2*, did not differ significantly between control and heat-stressed seeds (Fig. 2a–c), suggesting that GA biosynthesis has less effect on delayed germination. Among ABA biosynthesis genes, the expression of *OsNCED1* fluctuated during germination, with no significant difference between treatments (Fig. 2d). The expression of *OsNCED4* increased after imbibition and was higher in the control than in heat-stressed embryos (Fig. 2g). The expression of *OsNCED2*, *OsNCED3*, and *OsNCED5* dropped dramatically upon imbibition from 6 to 18 HAI, suggesting their roles in seed germination (Fig. 2e,f,h). The expression of *OsNCED2* was similar in control and heat-stressed embryos at 6 HAI, but was significantly higher in heat-stressed embryos at the later time points (4.7, 2.4 and 3.0-fold at 12, 18 and 24 HAI, respectively; Fig. 2e). Similarly, *OsNCED5* expression was significantly higher in heat-stressed than in control embryos (7.6, 3.3 and 2.1-fold at 12, 18 and 24 HAI, respectively; Fig. 2h).

We also analysed transcriptional expression of three ABA catabolism genes. Upon imbibition, *OsABA8′OH1* expression was down-regulated in heat-stressed embryos, and the difference from the control was significant at 6, 12, and 24 HAI (Fig. 2i). Significant down-regulation of expression in heat-stressed embryos was also observed at 6 HAI for *OsABA8′OH2* (Fig. 2j), and from 6 to 18 HAI for *OsABA8′OH3* (Fig. 2k). As a result of higher expression of ABA biosynthesis genes and lower expression of ABA catabolism genes during imbibition, endogenous ABA content was significantly higher in heat-stressed in embryos at 6 and 12 HAI (Fig. 2l). Overall, these data suggest that higher endogenous ABA content in the embryo after imbibition was involved in delayed germination of heat-stressed seeds.

### Down-regulation of α-amylase gene expression in aleurone cells in response to exogenous GA

**Figure 3:**
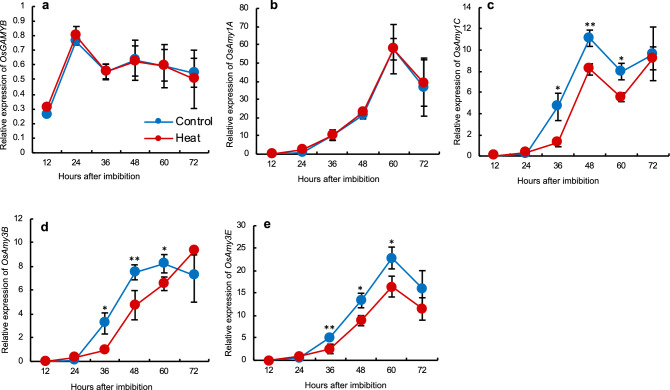
Expression of *GAMYB* transcription factor and α-amylase genes in embryoless imbibed seeds treated with exogenous 1 μM GA. (**a**) *OsGAMYB*; (**b**) *OsAmy1A*; (**c**) *OsAmy1C*; (**d**) *OsAmy3B*; (**e**) *OsAmy3E.* Significant differences are shown as *P* < 0.05* and *P* < 0.01** according to Student’s *t*-test (*n* = 3). Error bars, SD.

We also analysed transcriptional changes in aleurone cells during seed imbibition in response to GA. The expression of *OsGAMYB*, a GA-responsive transcription factor, in embryoless half-seeds imbibed with 1 μM GA changed similarly with time in control and heat-stressed seeds, with a peak at 24 HAI regardless of heat treatment (Fig. 3a). We measured the expression levels of *OsAmy1A*, *OsAmy1C*, *OsAmy3B*, and *OsAmy3E* because theses α-amylase genes are highly expressed in rice endosperm after seed imbibition^41^. *OsAmy1A* expression increased gradually, with the same pattern in control and heat-stressed seeds (Fig. 3b). The expression of *OsAmy1C*, *OsAmy3B*, and *OsAmy3E* was significantly lower in heat-stressed than in control seeds at 36–60 HAI (Fig. 3c–e), i.e., later than the *GAMYB* peak. These data suggest that down-regulated α-amylase gene expression in the aleurone layer contributed to the delayed germination of heat-stressed seeds.

### DNA methylation at the promoters of ABA catabolism-related and α-amylase genes in heat-stressed seeds

**Figure 4:**
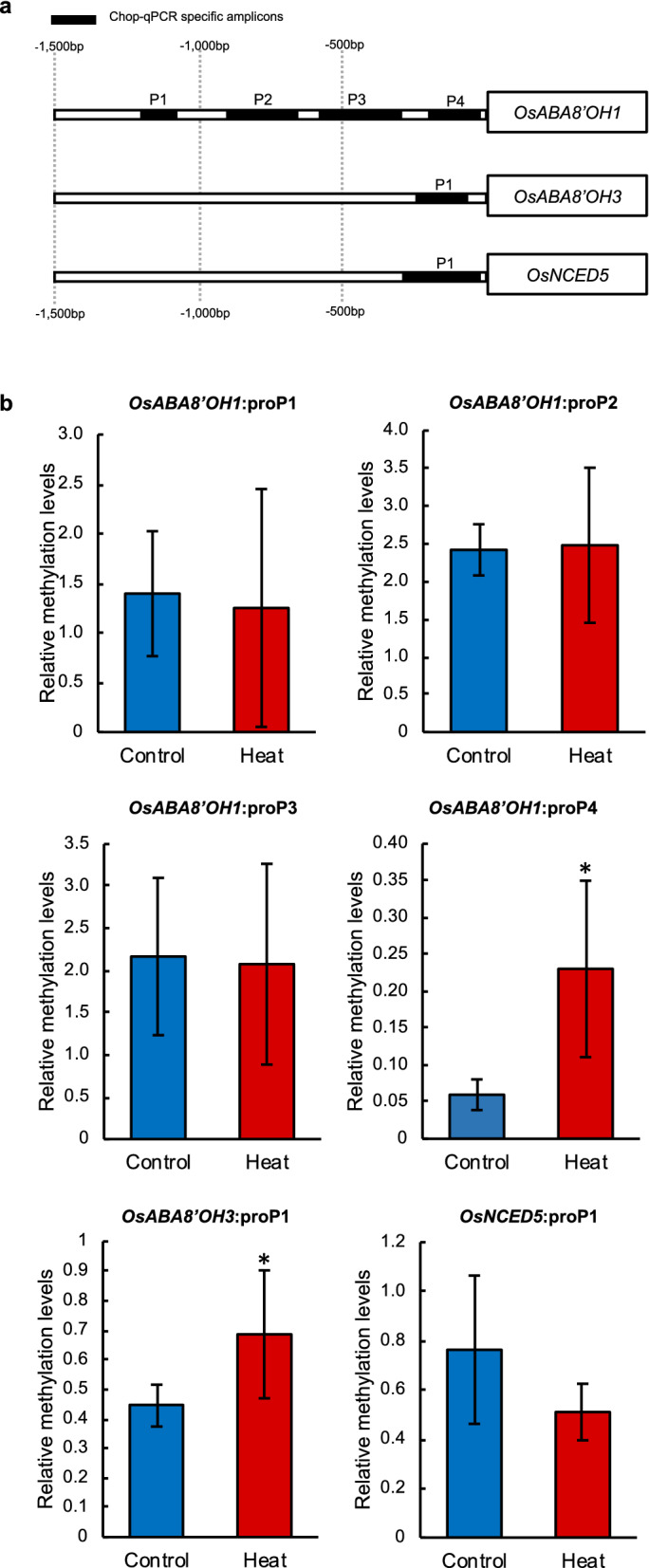
Relative methylation levels of *OsABA8′OH1, OsABA8′OH3* and *OsNCED5* promoter regions measured by Chop-qPCR. (**a**) Map of PCR amplicons of CpG islands predicted by MethPrimer. (**b**) Relative DNA methylation levels of *OsABA8′OH1*, *OsABA8′OH3*, and *OsNCED5* promoter regions in after-harvest control and heat-stressed seeds. Significant differences are shown as *P* < 0.05* according to Student’s *t*-test (*n* = 6).

**Figure 5:**
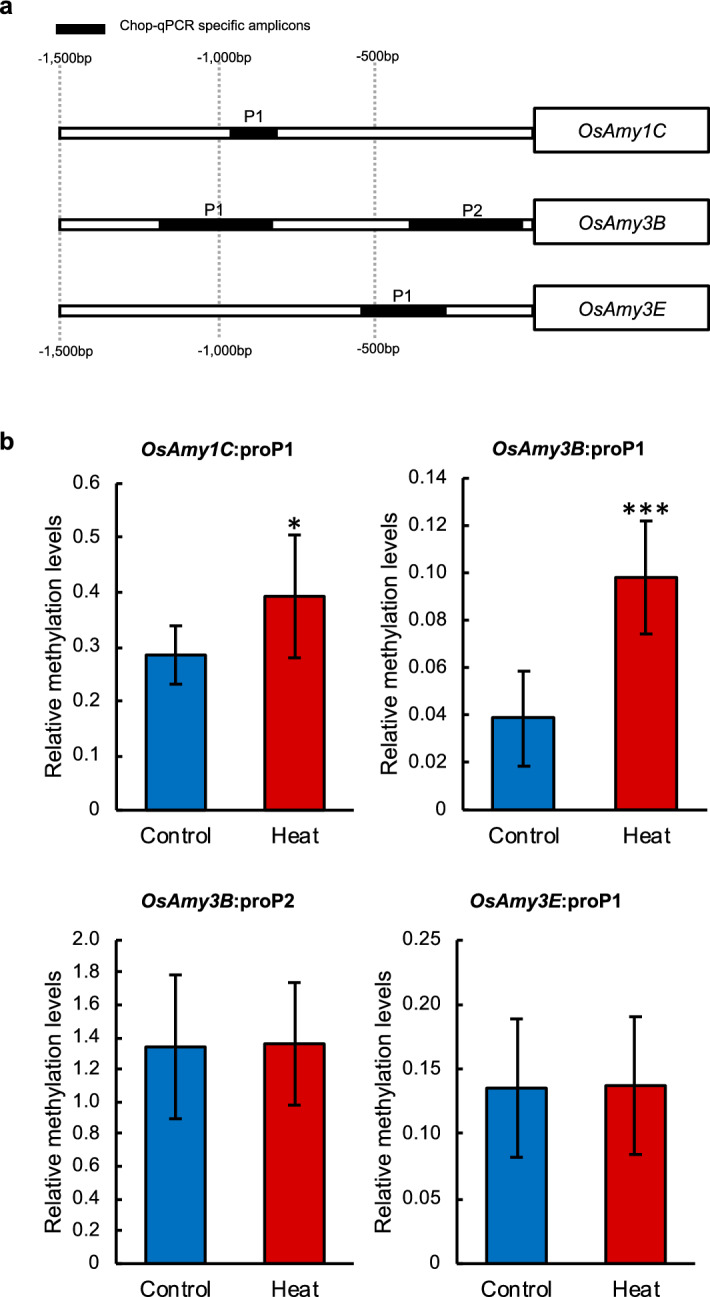
Relative methylation levels of α-amylase promoter regions measured by Chop-qPCR. (**a**) Map of PCR amplicons of CpG islands predicted by MethPrimer. (**b**) Relative DNA methylation levels of *OsAmy1C*, *OsAmy3B*, and *OsAmy3E* promoter regions in dry control and heat-stressed seeds. Significant differences are shown as *P* < 0.05* and *P* < 0.001*** according to Student’s *t*-test (*n* = 6). Error bars, SD.

To test whether epigenetic changes affect the expression of the above genes, we analysed their promoters for possible DNA methylation regions. We found predicted CpG islands in the promoters of the ABA metabolism genes *OsNCED5*, *OsABA8′OH1*, and *OsABA8′OH3*, and of the α-amylase genes *OsAmy1C*, *OsAmy3B*, and *OsAmy3E*, but not in those of *OsNCED2*, *OsABA8′OH2*, or *OsAmy1A*. Relative methylation levels in the CG-context in dry seeds are shown in Fig. 4. The methylation level at *OsABA8′OH1*:proP4, the predicted region nearest to the transcription start site (Fig. 4a), in heat-stressed seeds was 3.8 times higher than that in control seeds (Fig. 4b). Methylation of the *OsABA8′OH3*:proP1 region was also significantly higher in heat-stressed than in control seeds, but that of the *OsNCED5*:proP1 region was lower but not significant (Fig. 4b). *OsAmy1C*:proP1 and *OsAmy3B*: proP1 regions showed significant hyper-methylation (1.3 and 2.5-fold, respectively) in heat-stressed seeds in comparison with the control (Fig. 5). Hyper-methylations of *OsABA8*′*OH1*:proP4, *OsABA8*′*OH3*:proP1, *OsAmy1C*:proP1 and *OsAmy3B*:proP1 promoters were also observed by using MeDIP-qPCR identifying methylation levels, suggesting the similar results (Supplemental Fig. 5). The methylation levels of *OsAmy3B*:proP2 and *OsAmy3E*:proP1 were not affected by heat treatment. Thus, heat stress significantly increased methylation levels in promoters of some genes.

## Discussion

We found that heat stress during grain filling significantly delayed seed germination, but that this phenomenon was not due to primary dormancy. Germination of the seeds of Nipponbare and the other rice cultivars tested (one heat stress-sensitive and five tolerant) was delayed to different extents by heat stress during grain filling. Under heat stress, sensitive cultivars develop more grain chalkiness than do tolerant cultivars^8^. Therefore, this phenomenon is not cultivar specific and is not associated with the level of grain chalkiness under heat stress.

High temperature during maturation increases rice seed dormancy^26,27^. After harvest rice seed dormancy is broken by high temperature^28,29^, and after-ripening^30,31^ are commonly used to release seed dormancy. In this study, the dormancy levels of both control and heat-stressed seeds were significantly decreased after dormancy release by dormancy-break or after-ripening treatment. Yet heat-stressed seeds still germinated significantly slower than control seeds. Therefore, the delay in germination by exposure to high temperature during maturation is not due to primary dormancy. *Delayed of germination1* (*DOG1*), is a well-known master key controlling primary dormancy through maternal environmental factors during seed maturation^32^. Although of some rice japonica-type DOG1-like genes^33^ were up-regulated during grain filling stage under heat stress (Supplemental Fig. 3a), a previous study in *Arabidopsis thaliana* shows that after-ripening treatment changes the protein structure of DOG1 and abolishes its activity^34^. Also, *OsDOG1-like* gene expression in imbibed embryos was not strongly induced or inhibited by heat stress during grain filling (Supplemental Fig. 3b). Taking together, primary dormancy and DOG1 were not involved in delayed germination of heat stressed seeds.

During seed imbibition, GA content increases^14^ and ABA content drops dramatically^35^ to allow germination. In imbibed embryos, the expression of the GA biosynthesis genes *OsGA3ox2*, *OsGA20ox1*, and *OsGA20ox2* gradually increased toward germination. Os*GA3ox2* is highly expressed during seed germination to induce the expression of α-amylase, whereas the expression of its homolog, *OsGA3ox1*, is lower^14^. *OsGA20ox1* and *OsGA20ox2* are involved in GA biosynthesis during seed germination^36,37^. In this study, no difference in GA biosynthesis gene expression was observed, but higher expression of ABA biosynthesis genes and lower expression of ABA catabolism genes were observed in heat-stressed seeds. *OsNCED2* and *OsABA8′OH3* play predominant roles in ABA metabolism in imbibed rice seeds^38^. The changes in the transcription of these genes in embryos during imbibition resulted in a significantly higher ABA content in heat-stressed seeds. At the very early phase of imbibition, control and heat-stressed seeds had the same ABA levels. However, the ABA levels were significantly higher in heat-stressed embryos at later time points. GAMYB is a GA-responsive transcription factor essential for α-amylase induction in the aleurone^39^. A GARE box in the α-amylase promoter is essential for direct binding of GAMYB and for transcriptional induction^40^. We showed that the *OsGAMYB* gene expression level was indistinguishable between control and heat-stressed seeds, indicating that the response to exogenous was not affected by heat stress during grain filling. Among eight rice α-amylase isozymes, promoters of genes encoding OsAmy1A, OsAmy1C, OsAmy3B, and OsAmy3E contain GARE boxes^41^. The expression of α-amylase genes started to increase after the *GAMYB* expression had peaked. Among the α-amylase genes examined, *OsAmy1A* was particularly highly induced by GA in the aleurone. *OsAmy1A*, the most predominant GA responsive gene, showed no difference between the two treatments, while *OsAmy1C*, *OsAmy3B*, and *OsAmy3E* expression was significantly lower in heat-stressed than in control seeds at 36–60 HAI. This might result in non-drastic delayed seed germination. These observations suggest that changes in the expression of ABA metabolism genes in the embryo and of α-amylase genes in the aleurone layer after imbibition delayed germination of heat-stressed seeds.

DNA methylation is a well-known epigenetic response for gene regulation, including silencing^42^. A recent study has shown that DNA methylation in the promoter of the gene for ALLANTOINASE, a negative regulator of dormancy, is stimulated by cold experienced by the mother plant and is passed to the seeds^43^, suggesting that DNA methylation in the mother plant is important for seed germination. We found that *OsABA8*′*OH1* and *OsABA8*′*OH3* promoters were highly methylated and the *OsNCED5* promoter tended to be hypo-methylated in heat-stressed seeds, which corresponded to the lower expression of the former two genes and higher expression of *OsNCED5*. Promoter analysis revealed that only *OsAmy1A* contained no predicted CpG island for DNA methylation, at least within − 1500 bp, whereas *OsAmy1C* and *OsAmy3B* promoters were highly methylated in heat-stressed seeds and their methylation levels corresponded to down-regulation of gene expression. The presence of CpG islands and hyper-methylation in α-amylase promoters play a role in heat stress-induced transcriptional regulation during seed imbibition. In conclusion, we propose that heat stress during grain filling changes the expression of genes involved in ABA metabolism and α-amylase genes during imbibition via DNA methylation of the respective gene promoters, delaying seed germination.

Other factors such as histone modifications are involved in epigenetic regulation. In *Arabidopsis*, the *HUB1* (*HISTONE MONOUBIQUITINATION 1*) gene is involved in H2B monoubiquitination, which regulates the methylation of this histone^44^, and the *hub1* mutant shows reduced expression of *NCED9* and *ABI4* (*ABSCISIC ACID INSENSITIVE4*) but increased expression of *ABA8*′*OH2*^45^. In *Arabidopsis*, acetylation of H3K9K19 also regulates *ABA8′OH* expression and seed dormancy during maturation^46^. In rice, H3K9 deacetylation by *OsSRT1* (*Sirtuin1*) mediates starch metabolism genes such as *OsAmy3B*, *OsAmy3E*, *OsBmy4* and *OsBmy9*^47^.

These data suggest that other epigenetic changes together with DNA methylation might regulate germination in heat-stressed seeds.

## Materials and methods

### Plant materials and growth conditions

Three-week-old seedlings of rice (*Oryza sativa* L.) ‘Nipponbare’ were transplanted into 1/5000-a Wagner pots (10 plants per pot) with 8.75 g of basal dressing compound fertilizer (N–P_2_O–K_2_O: 4%–4%–4%) and 0.85 g of sigmoid-type controlled-release coated urea in 2015. In addition, 0.5 g of ammonium sulfate (N: 21%) was applied twice, at the tiller development stage and at the panicle booting stage. All fully developed tillers were removed and only the main stems were used in this study. Plants were grown under natural conditions at Kyushu University (33°67′N, 130°42′E) until spikelets located on the upper primary rachis branches flowered in more than 50% of the whole population. That day was set as the day of flowering (0 days after flowering: DAF) and the plants were transferred into two different temperature regimes (25 °C, control; or 30 °C, heat treatment) in a growth chamber with natural light until harvest at 42 DAF. Harvested seeds were dried at room temperature for 1 week and stored at − 0 °C to maintain seed dormancy. Same cultivation methods were also repeated in 2016 and 2017. In some experiments, seeds were incubated at 45 °C (dormancy-break treatment) for 3, 5, 7, 10 and 14 days, or at 26 °C (after-ripening treatment) for 2, 4, 6, 8, 10 weeks in the dark prior to germination tests.

### Germination test

Seeds were rested at room temperature for 1 h and sterilized with 0.2% NaClO for 20 min. Seeds were allowed to germinate on filter paper in 9-cm Petri dishes filled with 10 mL distilled water at 27 °C in the dark. One Petri dish contained 30 seeds (one replication). Germination rates (emergence of 1-mm shoots) were checked every 12 h. The *T*_50_ (the average time to reach 50% germination) of each sample was calculated^48^.

### RNA extraction and quantitative real-time PCR

Seeds were germinated as above and embryos from each treatment group were sampled at 6, 12, 18, or 24 h after imbibition (HAI). Embryoless half-seeds were placed upside down in Petri dishes with filter paper soaked with 6 mL of 1 μM GA and were sampled at 12, 24, 36, 48, 60, and 72 HAI. Total RNA from embryos and embryoless half-seeds was extracted from frozen materials using the SDS/phenol/LiCl method^49^. cDNA was synthesised from extracted RNA using ReverTra Ace reverse transcriptase (Toyobo) according to the manufacturer’s instructions. Quantitative real-time PCR was performed using a MiniOpticon real-time PCR detection system (Bio-Rad) with SYBR Green (Toyobo) as described in the manufacturers’ instructions. Primers used for qRT-PCR are listed in Table S1. PCR thermal cycling conditions were as follows: initial denaturation, 94 °C for 2 min; 40 cycles of denaturation 94 °C for 20 s, annealing at primer-specific temperature for 20 s, and extension at 72 °C for 20 s; followed by melting and plate reading. The results were normalized to the expression level of the *OsActin* gene.

### Endogenous ABA content in embryos

Embryos isolated from 30 imbibed seeds (1 replicate; 30–50 mg) at 3, 6, and 12 HAI were crushed thoroughly with a pestle in a mortar with 2 mL 80% methanol and centrifuged at 10,000 rpm at 4 °C for 20 min. Supernatants were evaporated overnight. ABA content was measured as absorbance at 405 nm using a Phytodek competitive ELISA kit (Agdia) according to the manufacturer’s instructions.

### DNA methylation analysis by Chop-qPCR

Genomic DNA from 0.1 g dry seeds was extracted using a DNeasy Plant Maxi Kit (Qiagen) as described in the manufacturer’s instructions. Genomic DNA (0.5 μg) was digested (total reaction volume, 20 μL) with the methylation-sensitive restriction enzyme^50^ *Hpa*II (New England Biolabs) at 37 °C for 1 h, followed by enzyme inactivation at 80 °C for 20 min. Digested DNA (25 ng) was subjected to qRT-PCR using SYBR Green (Toyobo). Relative methylation levels were calculated from ΔCt values and were standardized to undigested control DNA^50^. Specific primer sets (listed in Supplemental Table 1) were designed for putative methylation regions predicted in MethPrimer software (https://www.urogene.org/methprimer/).

### DNA methylation analysis by MeDIP-qPCR

Genomic DNA from 40 dry seeds per sample was extracted using DNeasy Plant Kit (Qiagen) and was sheared to about 500 bp by sonication. Sheared DNA was immunoprecipitated using MagMeDIP Methylated DNA Immunoprecipitation Kit as described by the manufacturer’s protocol and a previous study applied to plant samples^51^. Immunoprecipitated DNA was subjected to qRT-PCR using SYBR using the same specific primers as Chop-qPCR. Percent recovery was calculated as described in manufacturer’s protocol (% recovery = 2^[Ct(10% input) − 3.32 − Ct(IP sample)] × 100).

All data generated or analysed during this study are included in this published article and its “Supplementary information” files.

## Supplementary information

Supplementary Information.
