# Supplementary material for: Mechanism of delayed seed germination caused by high temperature during grain filling in rice (Oryza sativa L.)
Source: Sci Rep. 2020 Oct 15;10:17378. doi: 10.1038/s41598-020-74281-9 (PMC7562956; doi:10.1038/s41598-020-74281-9)
Supplement: Supplementary file 1 — Supplementary Information. [file 41598_2020_74281_MOESM1_ESM.pdf]

Mechanism of Delayed Seed Germination Caused by High Temperature During Grain Filling in Rice (*Oryza sativa* L.)

Chetphilin Suriyasak[1], Yui Oyama[1], Toshiaki Ishida[2], Kiyoshi Mashiguchi[2], Shinjiro Yamaguchi[2], Norimitsu Hamaoka[1], Mari Iwaya-Inoue1, Yushi Ishibashi[1]*

[1]Faculty of Agriculture, Kyushu University, Fukuoka, Japan
[2]Kyoto University, Kyoto, Japan

Supplemental Table 1

Supplemental Figure 1

Supplemental Figure 2

Supplemental Figure 3

Supplemental Figure 4

Supplemental Table 1 Primer sequences used for qRT-PCR and Chop-qPCR

qRT-PCR primers	Forward (5’-3’)	Reverse (5’-3’)
OsActin	GACTCTGGTGATGGTGTCAGC	GGCTGGAAGAGGACCTCAGG
OsGA3ox1	GGAGAGCAAGGCCGTGTATCAGG	CTCTCCTTGTCCTCTTCCTTCGCTAC
OsGA20ox1	CCACTACTTCCGGCGATTCTTCCAGCG	GACGTGGTCCTGGTGGAGGATGGTG
OsGA20ox2	CGCCGACTACTTCTCCAGCACC	GCTGTCCGCGAAGAACTCCCT
OsNCED1	CTGGAGCACATGGAGCTAGTGCACTCC	CCGACGCCGAAGTAGCCGTACCTG
OsNCED2	TCATTCCAAAACACCTTCCA	TCCGGGGACCTCCTATGTAT
OsNCED3	CCCCTCCCAAACCATCCAAACCGA	TGTGAGCATATCCTGGCGTCGTGA
OsNCED4	TCCATCTCCTTCTCCCTCCTCCC	CCTCGCACCCTGCTTGATCTTGCC
OsNCED5	CACATCCGAGCTCCTCGTCGTGAAC	GAATCAAGAATCCATGGATGGCCGGTG
OsABA8’OH1	AAGTACAGGTGGTCCACGTCCAA	CCAGCTTAGCTGATGCTAGTATTC
OsABA8’OH2	GACGAGGTGGAGTACAGCCCGTTC	GGACACATCAGCCACCATCAGCAGTAG
OsABA8’OH3	AGTACAGCCCATTCCCTGTG	ACGCCTAATCAAACCATTGC
OsGAMYB	CAGTGGCAATTCATTCACTGAATC	TCCAGATCCCATTGAAGTGCTTTG
OsAmy1A	GATACGACGTCGAACACCTC	CGGATCGGATACAGCTCGTTG
OsAmy1C	TATCATGGAGGCTGACAGCG	GCTAATTGTGCCTCTCCACC
OsAmy3B	GATTGGGACACGGTATGACG	CTGCAGGAACTCTGAGACCG
OsAmy3E	GAGAAGGAAGGCCTCAGGGTTCCTG	GAAGCTCGCTCGTACACATCTCGCAG
OsDOG1L1	GAGTCGTTGGCGACGGTGAAG	ATGAGGAAGTGCACGGCCTG
OsDOG1L2	TCTTAGTGAGTGGTGTGCTGGGA	GTGAATCATCAGCTTTGCACAGC
OsDOG1L3	CAGTAGCAATAGTACCTCATAAT	AGCGAATCATGATTGTGACCTG
OsDOG1L5-1	GACAATCCGACAAGCTGCCC	GTTGTTACTCCCGTGGCCTA
OsDOG1L5-3	TCCTGAGAGAGCGTTAACTGCG	TACAGGCCCAAGAGCCCAATCTA

Chop-qPCR primers	Forward (5’-3’)	Reverse (5’-3’)
OsABA8’OH1: proP1	GAGCTGCATGCGCACGACCATTTC	CGGTATCACTGACCGCGGCTAGC
OsABA8’OH1: proP2	AGTACGTAGTACGTACGAGAGCGC	TACAGTGATAGCTAAATGGGCCTC
OsABA8’OH1: proP3	ATTGATTGCGCAATTATTCGGCAG	TACTAGTAGCTAGCACCACCACTA
OsABA8’OH1: proP4	GTGCTCGCGCCTTTCTTGCTTGT	GGTTCTTGGACGAGTACAGCTGGA
OsABA8’OH3: proP1	CCTAGCTCGCGGACACCATATGAG	GATCTTTCGAATGGTGGTGGGCGT
OsNCED5: proP1	TGTCTCCTTCCTCCCCCAAACTC	AGGAGAGGTGAGAGCTGGAGTTGG
OsAmy1C: proP1	GTACATGAACAGAGGTCGCAATGC	AACAGATTTTGCTAGTCGGTAGTC
OsAmy3B: proP1	CGCTGGATGCGGTGCTCGTTGAAG	GCACCGCCGAACTCGACGACCAGC
OsAmy3B: proP2	GCTGCATAGCACGACGCATACATA	ACTTGGCCAATGGATGGATCGTTG
OsAmy3E: proP1	GCGCTTGATCCGTCACACCGATGA	ACGCATGACGGAAACGGCGATACG

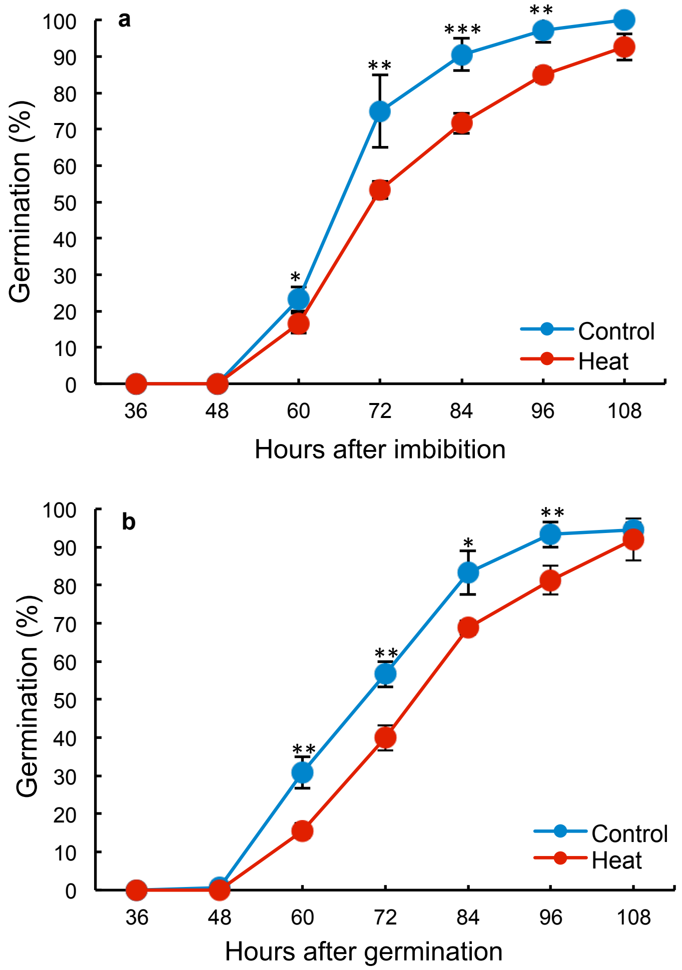

Supplemental Figure 1. Delay in germination caused by heat stress during grain filling in **a,** 2016 (n = 4) and **b,** 2017 (n = 3) with 30 seeds per replicate. Seeds were germinated at 27°C in the dark. Significant differences are shown as P < 0.05*, P < 0.01**, and P < 0.001*** according to Student's t-test. Error bars, SD.

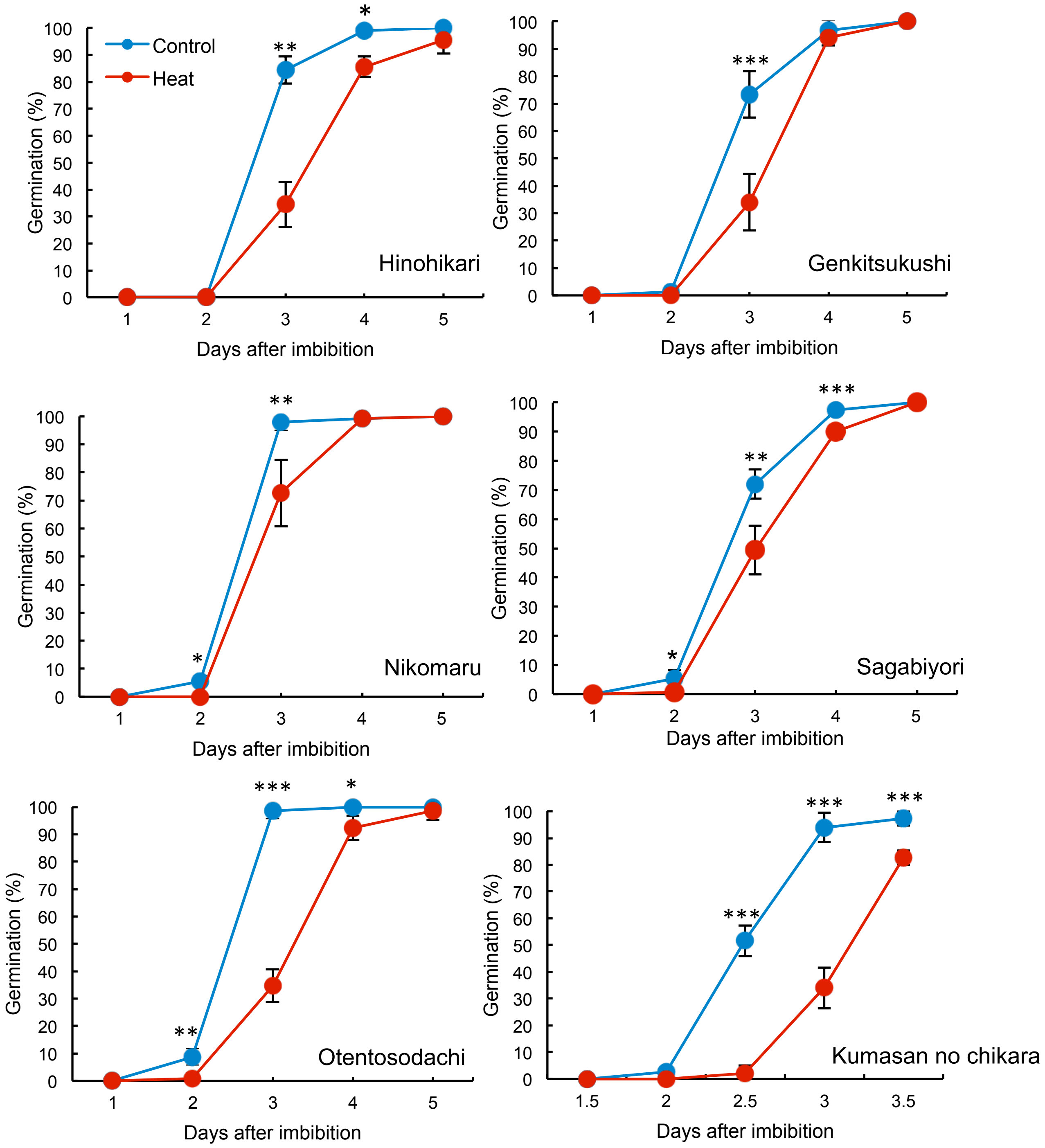

Supplemental Figure 2. Delay in germination of heat-stressed seeds of the heat stress–sensitive rice cultivar 'Hinohikari' and heat stress–tolerant cultivars 'Genkitsukushi', 'Nikomaru', 'Sagabiyori', 'Otentosodachi', and 'Kumasan no chikara'. Harvested seeds were obtained from Tanamachi et al. (2016), who used exactly the same cultivation methods as in this study. Seeds were germinated at 27°C in the dark. Significant differences are shown as $P < 0.05$*, $P < 0.01$**, and $P < 0.001$*** according to Student's *t*-test ($n = 3$). Error bars, SD.

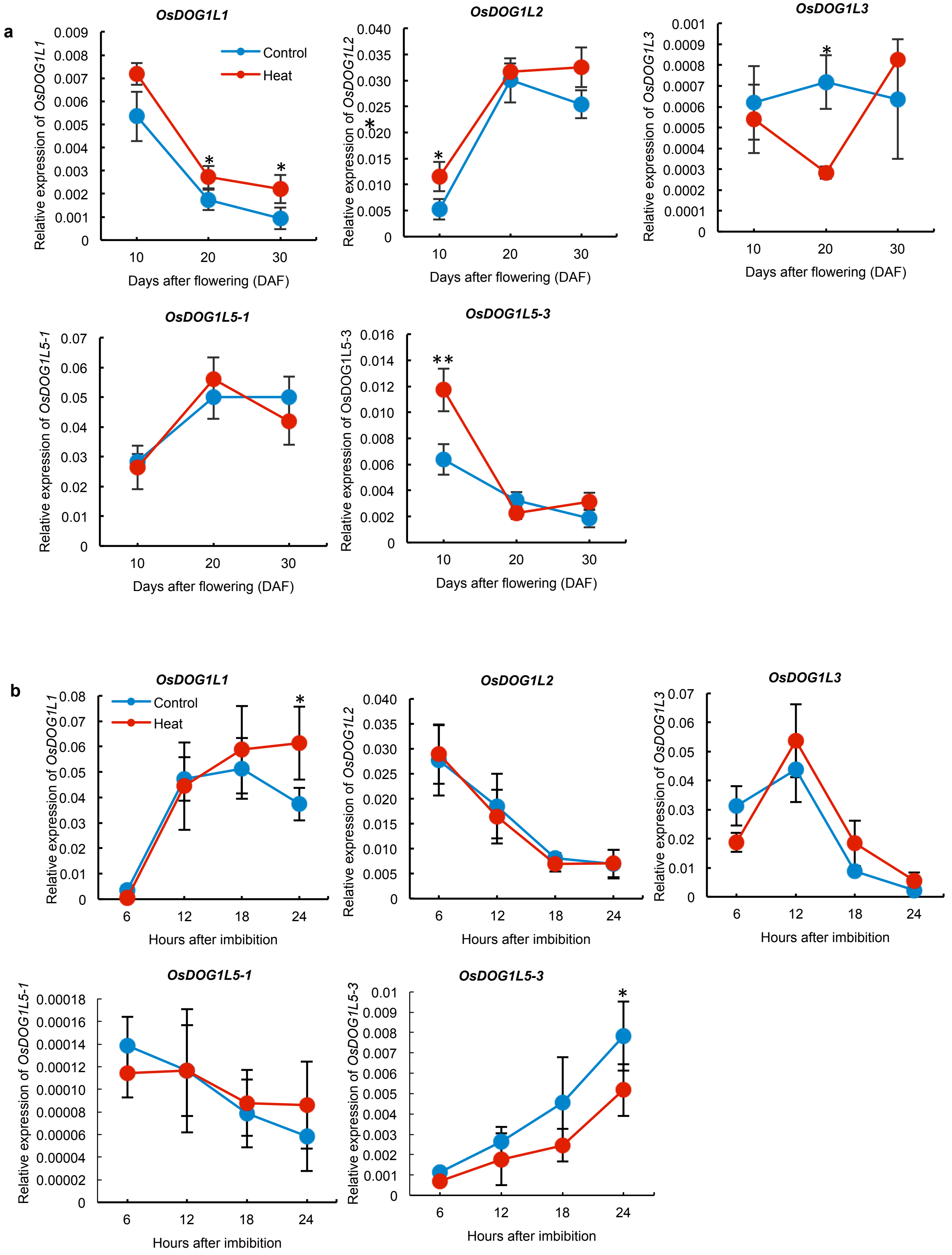

Supplemental Figure 3. Expression of DOG1-like genes in control and heat. a. Relative gene expression of *DOG1-like* genes in developing seeds under control (25°C) and heat (30°C) conditions. **b.** Relative gene expression *of DOG1-like* genes in embryos during imbibition. Significant differences are shown as $P < 0.05$*, $P < 0.01$**, and $P < 0.001$*** according to Student's *t*-test ($n = 3$). Error bars, SD.

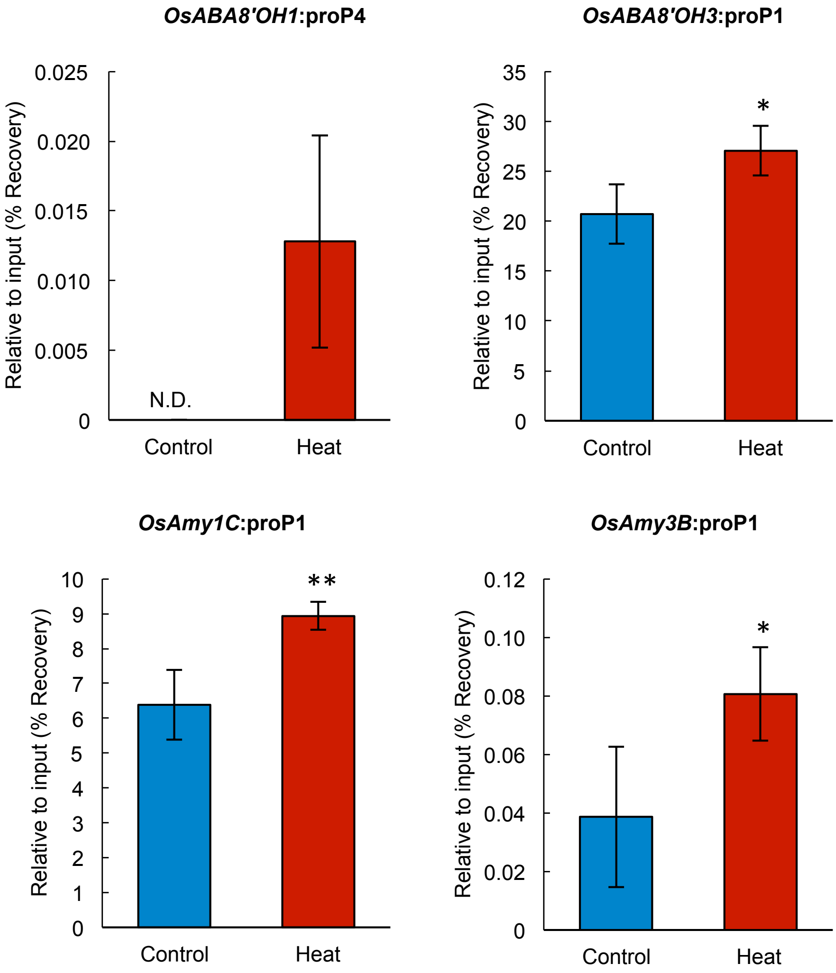

Supplemental Figure 4. DNA methylation levels of *OsABA8'OH1*, *OsABA8'OH3*, *OsAmy1C* and *OsAmy3B* promoters. Methylated DNA was precipitated and analyzed via MeDIP-qPCR. Significant differences are shown as $P < 0.05$* and $P < 0.01$** according to Student's *t*-test ($n = 3$). Error bars, SD.
